# Locking retrograde nail, non-locking retrograde nail and plate fixation in the treatment of distal third femoral shaft fractures: radiographic, bone densitometry and clinical outcomes

**DOI:** 10.1186/s10195-021-00593-9

**Published:** 2021-08-04

**Authors:** Luigi Meccariello, Michele Bisaccia, Mario Ronga, Gabriele Falzarano, Auro Caraffa, Giuseppe Rinonapoli, Predrag Grubor, Valerio Pace, Giuseppe Rollo

**Affiliations:** 1 Department of Orthopedics and Traumatology, AORN San Pio, Via Cupa dell’Angelo, Block: Moscati Floor:2, 82100 Benevento, Italy; 2 Division of Orthopedics and Trauma Surgery, University of Perugia, “S. Maria Della Misericordia” Hospital, Perugia, Italy; 3 Orthopaedic and Trauma Operative Unit, Department of Biomedical and Dental Sciences and Morpho-Functional Imaging, University of Messina, University Hospital G. Martino, Messina, Italy; 4 Department of Orthopedics and Traumatology, Azienda Ospedaliera “Gaetano Rummo”, Benevento, Italy; 5 School of Medicine, University of Banja Luka, Banja Luka, Bosnia and Herzegovina; 6 The Royal National Orthopaedic Hospital, Stanmore, London, UK; 7 Department of Orthopedics and Traumatology, Vito Fazzi Hospital, Lecce, Italy

**Keywords:** Distal femur shaft fractures, Locking plate, Retrograde nail, Bone healing, Radiographic assessment

## Abstract

**Background:**

Distal third femoral shaft fractures are characterized by increasing incidence and complexity and are still considered a challenging problem (high morbidity and mortality). No consensus on best surgical option has been achieved. This study aims to investigate radiographic, mineral bone densitometry and clinical outcomes of locking retrograde intramedullary (LRN) nailing, non-locking retrograde intramedullary nailing and anatomical locking plate to surgically treat distal third femoral shaft fractures in young adults. Our hypothesis was that there is no significant statistical difference among the surgical options in terms of results (radiographic, bone densitometry and outcomes assessment).

**Methods:**

Retrospective study: 90 patients divided into three groups (group 1 LRN, group 2 NLRN, group 3 plating). Average age was respectively 42.67 (± 18.32), 44.27 (± 15.11) and 42.84 (± 18.32) years. Sex ratio F:M was respectively 2.75, 2.33 and 2.00. AO Classification, KOOS, NUSS and RUSH score, VAS, DEXA scans and plain radiographs were used. Evaluation endpoint: 12 months after surgery.

**Results:**

There were no statistical differences in terms of surgery time, transfusions, and wound healing. Results were similar with regard to average time of bone healing, RUSH scores, VAS, KOOS, regression between RUSH and VAS, average correlation clinical–radiographic results and patients outcomes.

**Conclusions:**

Our results showed no statistical difference in the use of LNR, NLNR and plating for treatment of distal third femur shaft fractures in terms of radiographic, bone densitometry and clinical outcomes. Good subjective and objective results are provided by all three techniques. The choice among the studied techniques must be based on surgeons’ experience, indications and subjective patients’ aspects. The absence of relevant similar data in the published literature does not allow definitive validation (or rejection) of our hypothesis. A more powered study with a bigger cohort is needed for definitive validation.

## Introduction

Distal third femoral shaft fractures are a pattern of injury that currently accounts for about 4–6% of all femur fractures. The incidence and complexity are increasing due to the increasing rate of high-energy trauma, particularly in young patients. In contrast, low-energy fractures (on native or prosthetic knee) of osteoporotic bones are more characteristic in the elderly population [1]. There are a number of reasons why these fractures remain a challenging problem for the orthopaedic surgeons, including high complication rates, significant associated morbidity and mortality, number of non-union and delayed union cases, duration of rehabilitation, duration of surgery, blood loss and strong impact on quality of life [2].

Several conservative and surgical strategies have been studied with various and controversial results. Studies have shown that internal fixation devices (both anterograde and retrograde nails) provide superior outcomes as compared with other techniques by providing good stability at the fracture site that subsequently allows early mobilization, together with reduction in surgical blood loss, operating time and hospitalization compared with other more invasive procedures. Other surgical options include the use of locking plates, cannulated screws, external fixation, blade plates and distal femoral replacement. Recent studies have reported the possibility of proximal not locking retrograde nail to fix distal third femoral shaft fractures [3, 4]. This could potentially become one of the most frequently used surgical techniques. However, no specific studies have been carried out yet, and no comparisons with other techniques have been reported. Therefore its potentials, indications and results are not yet clearly known. To the best of our knowledge, no study exists that compares results of locking retrograde nailing and not locking retrograde nailing in the treatment of distal third femur fractures. The same applies to studies comparing the use of locking retrograde nails, non-locking retrograde nails and plating procedures [5–7].

This study aims to investigate and compare results in terms of radiographic, mineral bone densitometry and clinical outcomes of locking retrograde intramedullary nailing (RLN), non-locking retrograde intramedullary nailing (NRLN) and anatomical locking plate used to fix distal third femoral shaft fractures in young adults. Our hypothesis was that there is no statistical difference with regard to results.

## Materials and methods

Ninety patients were finally included in our study. They all sustained a distal third femoral shaft fracture. All patients were admitted, treated and followed up at three linked specialist trauma centres in the set time-frame of 5 years. These 90 patients were further divided into three groups: group 1 if treated with locking retrograde intramedullary nailing (RLN), group 2 if treated with non-locking retrograde intramedullary nailing (NRLN), or group 3 if treated with locking plating. Average age was respectively 42.67 (± 18.32), 44.27 (± 15.11) and 42.84 (± 18.32) years. Sex ratio F:M was respectively 2.75, 2.33 and 2.00.

Inclusion criteria: patients who sustained a distal third femoral shaft fracture (3.2 type according to the AO Classification System) [8] in the set time-frame of 5 years and were admitted and treated at three trauma centres by one of the three studied surgical options; age between 18 and 65 years; pre-trauma conditions and absence of local or systemic disease able to affect the surgical treatment and comorbidity and mortality; fitness to undergo surgery from the anaesthetic team; availability for 12 months post-operative clinical and radiological follow-up.

Exclusion criteria: haematological or oncological patients; presence of acute or chronic infections; 3.1 and 3.3 types of fracture according to the AO Classification System [8]; age under 18 or over 65 years for males and age over 50 years for females or early menopause patients; bone metabolism disorders; rheumatological diseases; polytrauma; previous injury on ipsilateral lower legs.

All fractures were classified according to the AO Classification [8]. Classifications for all patients and characteristics of the groups are described in Table 1.

**Table 1:** Description of the populations

Description of population	LRN	NLRN	Plate
Number of patients	30	30	30
Average age	42.67 (± 18.32)	44.27 (± 15.11)	42.84 (± 18.32)
Range of age	18–65	18–65	18–65
Sex ratio (female:male)	2.75 (22:8)	2.33 (21:9)	2.00 (20:10)
Mechanism of injury	Fall from height: 4 (13.33%)	Fall from height: 4 (13.33%)	Fall from height: 3 (10.00%)
	Traffic accident: 16 (53.34%)	Traffic accident: 18 (60.00%)	Traffic accident: 19 (63.33%)
	Work accident: 6 (20.00%)	Work accident: 7 (23.33%)	Work accident: 6 (20.00%)
	Shooting: 4 (13.33%)	Shooting: 1 (3.33%)	Shooting: 2 (6.66%)
AO Classification (3.2)	A1: 4 (13.33%)	A1: 3 (10.00%)	A1: 4 (13.33%)
	A2:4 (13.33%)	A2:4 (13.33%)	A2:5 (16.66%)
	A3:2 (6.67%)	A3:1 (10.00%)	A3:2 (10.007%)
	B1:4 (13.33%)	B1:6 (20.00%)	B1:5 (16.66%)
	B2:2 (6.67%)	B2:2 (6.67%)	B2:1 (3.33%)
	B3:4 (13.33%)	B3:6 (20.00%)	B3:5 (16.66%)
	C1:4 (13.33%)	C1:4 (13.33%)	C1:3 (10.00%)
	C2:4 (13.33%)	C2:3 (10.00%)	C2:4 (13.33%)
	C3:2 (6.67%)	C3:1 (3.33%)	C3:1 (3.33%)
Side	Right: 12 (40.00%)	Right: 14 (46.66%)	Right: 13 (43.33%)
	Left: 18 (60.00%)	Left: 16 (53.33%)	Left: 17 (56.66%)

All patients were informed in a clear and comprehensive way of the type of proposed treatments (see the Operative Surgical Technique sections below) and any other possible surgical and conservative alternatives. Patients were treated according to the ethical standards of the Helsinki Declaration, and were invited to read, understand and sign an informed consent form.

We retrospectively used the Non-Union Scoring System (NUSS) (Table 1) [9] to study the post-operative bone healing process on radiographs. The authors also utilized the Radiographic Union Score for Hip (RUSH) score provided by Chiavaras et al. [10, 11] and derived from the Radiographic Union Scale in Tibial Fractures (RUST) scoring system. RUSH provides four components of scores: cortical bridging, cortical disappearance, trabecular consolidation and trabecular disappearance. Each component can be scored from 1 to 3. Similarly, the two trabecular indices were scored from 1 to 3, each based on consolidation for one of the indices, and fracture line disappearance for the other. The overall RUSH score therefore ranged from a minimum of 10 to a maximum of 30.

Pain visual analogue scale (VAS) score was collected on the same day of the post-operative radiographic follow-up with plain radiograph films [12]. The Knee Injury and Osteoarthritis Outcome Score (KOOS) was used to correlate function and quality of life of the patients.

The authors studied the mineral bone densitometry of the proximal part of the femur by performing DEXA scans for all patients in the post-operative period [13]. The femoral alignment was measured using plain radiographs [two projections, anteroposterior (AP) and lateral views] and correlated with clinical outcomes. The evaluation endpoint was set at 12 months after surgery.

### Group 1 surgical technique (RLN)

After having checked for associated fractures (e.g. fracture of the ipsilateral femoral neck), alignment, knee stability and limb length, patients were positioned supine on the radiolucent table. Routine prep and draping with a sterile bump under the knee were carried out. The anterior patellar transtendinous approach to the knee was used with knee kept in about 30° of flexion to avoid the action of the gastrocnemius in moving the distal fragment (incision from inferior pole of patella and tenotomy). Self-retainers, suction of synovial fluid and accurate haemostasis performed to improve visualization. A guide-wire was then inserted at the level of the centre of the intercondylar notch to the distal metaphysis under fluoroscopy check, followed by appropriate reaming. The reamer was then removed and replaced by a ball-tip guide-wire in the femoral canal that was pushed into the canal. Axial traction was then applied at 30° knee angle to achieve good fracture reduction. The guide-wire subsequently was pushed past the fracture site and 3 cm past the lesser trochanter under fluoroscopy check. A ruler was used to decide the nail length. A nail 1.5 below the size of the last used reamer was then inserted over the guide-wire and pushed past the fracture site till fluoroscopy confirmation of good positioning. Distal interlocking screws (as indicated) were positioned (most distal first) using bicortical drilling and fluoroscopy. The process was the same for the proximal interlocking screws (most proximal first, 34 or 36 mm screws). Confirmation of final good metalwork position and no rotation of the distal femur were obtained with fluoroscopy (AP and lateral radiographs) with knee extended and 90° of bending. Good range of motion of both knee and hip, limb length and rotation were checked. Appropriate irrigation and haemostasis were assured throughout the entire procedure. Closure in layers (starting with patellar tendon and paratenon) was performed and surgical dressing applied.

### Group 2 surgical technique (NRLN)

This procedure was performed identically to the one described for the RLN group (including positioning, equipment, fluoroscopy, surgical steps, irrigation, haemostasis and closure) with the only exception that the proximal locking was not performed. This is in fact the main difference among the two groups and what we are aiming to study with our work.

A radiograph of the uninjured contralateral limb was made for all cases in order to decide the correct length of the nail to be used in the pre-operative stage. All patients were given the same rehabilitation program protocol. This included early passive and assisted knee mobilization (on first or second post-operative day as pain allowed) and foot pump exercises; all patients had a post-operative X-ray check of the operated limb and achieved progressive weight bearing, based on stability of the fracture on the X-ray and clinical conditions. A personalized physiotherapy program was then continued with the aim to achieve early full weight bearing and full range of motion (ROM), always considering the post-operative stability of the fracture and subjective individual aspects of the patients.

### Surgical technique for group 3

After having checked for associated fractures (e.g. fracture of the ipsilateral femoral neck), alignment, knee stability and limb length, patients were positioned supine on the radiolucent table. Routine prep and draping with a sterile bump under the knee were carried out. A midline approach with extended lateral para-patellar arthrotomy was used. This was followed by exposure of the femoral condyles and subluxation of the patella to achieve good fracture site exposure. Periosteal elevation of the capsule was performed assuring preservation of the lateral collateral ligament. Next step was the achievement of fracture reduction by the use of pointed reduction forceps under direct visualization and fluoroscopy check and the aid of K-wires. A locking plate was then positioned sub-muscularly followed by fixation of the distal segment first, assuring that screw trajectory was parallel to the joint (position checked with fluoroscopy). Partially threaded or overdrilled fully threaded screws through the plate were used to provide interfragmentary compression. Two locking screws were used to ensure plate and alignment. Additional screws were then positioned appropriately. Fluoroscopy images were taken to ensure good metalwork position and absence of penetration through the intercondylar notch and check length, rotation and alignment. Flexion–extension reduction was achieved. Appropriate irrigation and haemostasis were assured throughout the entire procedure. Closure in layers was performed and surgical dressing applied.

### Statistical analysis

Descriptive statistics were used to summarize the characteristics of the study group and subgroups, including means and standard deviations of all continuous variables. The *t*-test was used to compare continuous outcomes. The *χ*^2^ test or Fisher’s exact test (in subgroups smaller than ten patients) were used to compare categorical variables. The statistical significance was defined as *p* < 0.05. We used Pearson correlation coefficient (*r*) to compare the predictive score of outcomes and quality of life. Mean ages (and their range) of the patients were rounded at the closest year. The predictive score of outcomes and quality of life and their ranges were approximated at the first decimal, while Pearson correlation coefficient was approximated at the second decimal (*r*).

## Results

The mean of follow-up was 17.32 (± 0.42; range 12–24) months for LNR, 16.10 (± 0.37; range 12–24) months for NLRN, 16.45 (± 0.47; range 12–24) months for plate, *p* > 0.05. Average age was respectively 42.67 (± 18.32), 44.27 (± 15.11) and 42.84 (± 18.32). Sex ratio F:M was respectively 2.75, 2.33 and 2.00.

The surgery lasted an average of 51.6 (± 17.6; range 29–76) min in LNR, 49.5 (± 21.8; range 36–83) min for NLRN and 62.7 (± 21.3; range 38–89) min for plate, *p* > 0.05.

The RBC IU of peri-operative transfusions in our patients was, on average, 2.8 (± 1.3; range 0–7) in LNR, 2.8 (± 1.9; range 0–6) for NLRN and 2.9 (± 1.5; range 0–6) for plate, *p* > 0.05.

In all three groups, patients demonstrated wound healing within 21 days. During the follow-up, we had no significant post-operative complications in any of the groups.

The average time of bone healing was 144.5 (± 15.9; 72–170) days after the surgery in LNR, 142.6 (± 14.0; 69–175) days for NLRN and 143.2 (± 14.1; 72–178) days for plate, *p* > 0.05.

On average day of bone healing, RUSH was 26.1 (± 2.3; range 24.0–30.0) points in LNR, 26.6 (± 2.7; range 23.2–30.8) in NLRN and 26.3 (± 2.7; range 23.2–29.7) in plate, *p* > 0.05.

On average day of bone healing, VAS was 2.4 (± 0.7; range 0–4) points in LNR, 2.3 (± 0.7; range 0–4) in NLRN and 2.2 (± 0.8; range 0–4) in plate, *p* > 0.05.

**Fig. 1:**
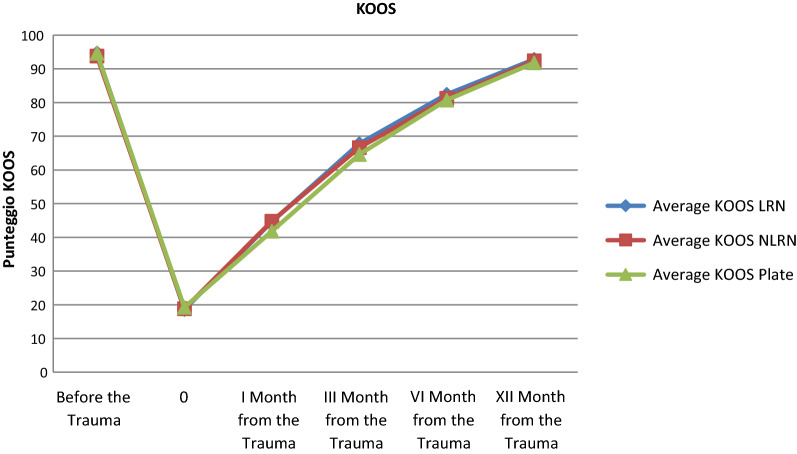
KOOS in the three groups. No statistically significant difference at the endpoint follow-up

The Knee Injury and Osteoarthritis Outcome Score (KOOS) was used to correlate function and quality of life. No significant difference was found, as shown in Fig. 1.

We found that, at average day of bone healing, the regression between RUSH and VAS scores showed a *p*-value of 0.057 in LNR, *p* = 0.062 in NLRN and *p* = 0.061 in plate.

Four patients had a reduction of mineral bone densitometry of proximal (from normal to ostheopenia) at the evaluation endpoints. Two belonged to group 1, one belonged to group 2 and the last one belonged to group 3.

The average correlation clinical–radiographic results and patients outcomes were high according Cohen *κ*: 0.859457333 ± 0.085103467 for LNR, *κ*: 0.823026667 ± 0.09557 for NLRN, *κ*: 0.853606667 ± 0.060782874 for plate, *p* > 0.05.

## Discussion

Fractures of the distal third of the femoral shaft are relatively rare (0.4% of all fractures and 3% of femoral fractures), with a bimodal distribution with a peak in frequency in young men (high energy) and elderly women (low energy). Conservative treatments are rarely used (reserved for bedridden patients or fractures with none or little displacement). A sufficient stabilization usually requires surgical management in order to withstand static and dynamic forces applied to the femur [10, 11].

These fractures are serious with a high mortality rate in elderly populations which is comparable to that found in the proximal femur. Most frequent complications include infection and septic non-union, aseptic non-union, residual stiffness and secondary post-traumatic osteoarthritis, with initial chondral injury as well as incomplete reduction [12]. Many surgical options are available for distal femur extra-articular fractures, including mini-invasive procedures. The primary goal is thought to be a good restoration of the articular surface, good reduction of femoral shaft fractures, maintenance of good stability and alignment, early mobilization and rehabilitation. The choice of the surgical technique takes into account patients’ functional goals, fracture characteristics, health comorbidities, bone quality, and risk of malunion and non-union [2].

Several studies have individually evaluated results of different surgical techniques (particularly intramedullary nails or plating procedures). Satisfactory results have been frequently achieved. However, controversies are still present; particularly, there is lack of well-defined and definitive guidelines on the use of every technique with regard to indications. Moreover, results vary significantly from one study to another [3, 4].

The traditional indications for the use of the nailing technique are the presence of an extra-articular fracture or a simple intra-articular fracture with little or no displacement [13]. The traditional indications for locking compression plating are the presence of an extra-articular fractures, sagittal unicondylar fractures or supra- and intercondylar fractures [14]. However, to the best of our knowledge, no studies have been performed to compare specifically locking retrograde intramedullary nailing, non-locking intramedullary nailing and plating procedures for the treatment of distal third femoral shaft fractures.

Several biomechanical studies have shown that locking systems are better than classic internal fixation (DCP plate, retrograde nailing, blade plate) [15–17]. Other studies have shown that LCP could cause higher complication, surgical revision and non-union rates, while in other studies this technique seemed to guarantee union in all cases, good recovery of alignment and high-quality function [18, 19]. Retrograde intramedullary nailing was shown to provide better surgical revision and malunion rates compared with other techniques [20–23].

Given this uncertainty about specific indications and related results, it seems that high-quality results are more dependent upon the surgical technique and experience of the surgeon than the choice of implant and that high-powered randomized multicentre studies are needed to achieve higher level of evidence with regard to surgical options to treat distal third femoral shaft fractures [24, 25].

We have therefore accordingly planned our study to compare results of the three studied surgical options. We present a retrospective group control study (30 patients forming each of the three groups) including patients treated at three linked trauma centres. We compared radiographic, bone densitometry and clinical outcomes of the three groups.

We did not find any significant difference in terms of duration of surgery between LNR and NLRN, despite the mean duration of LNR surgery exhibited a higher value (51.6 min against 49.5 min of NLRN) compared with NLRN. Slightly higher values were noted for the plating group, but this difference was related to implied technical surgical aspects of such procedures with no significant effects on results. Neither could we find a statistically significant difference with regard to the RBC IU of peri-operative transfusion (mean of 2.8 for LNR, 2.8 of NLRN, 2.9 for plate). All patients of all groups had appropriate wound healing by 21 days post-operation. These results show that both procedures imply similar risks for the patients related to duration of surgery, wound management and complications such as management of peri-operative blood loss.

**Fig. 2:**
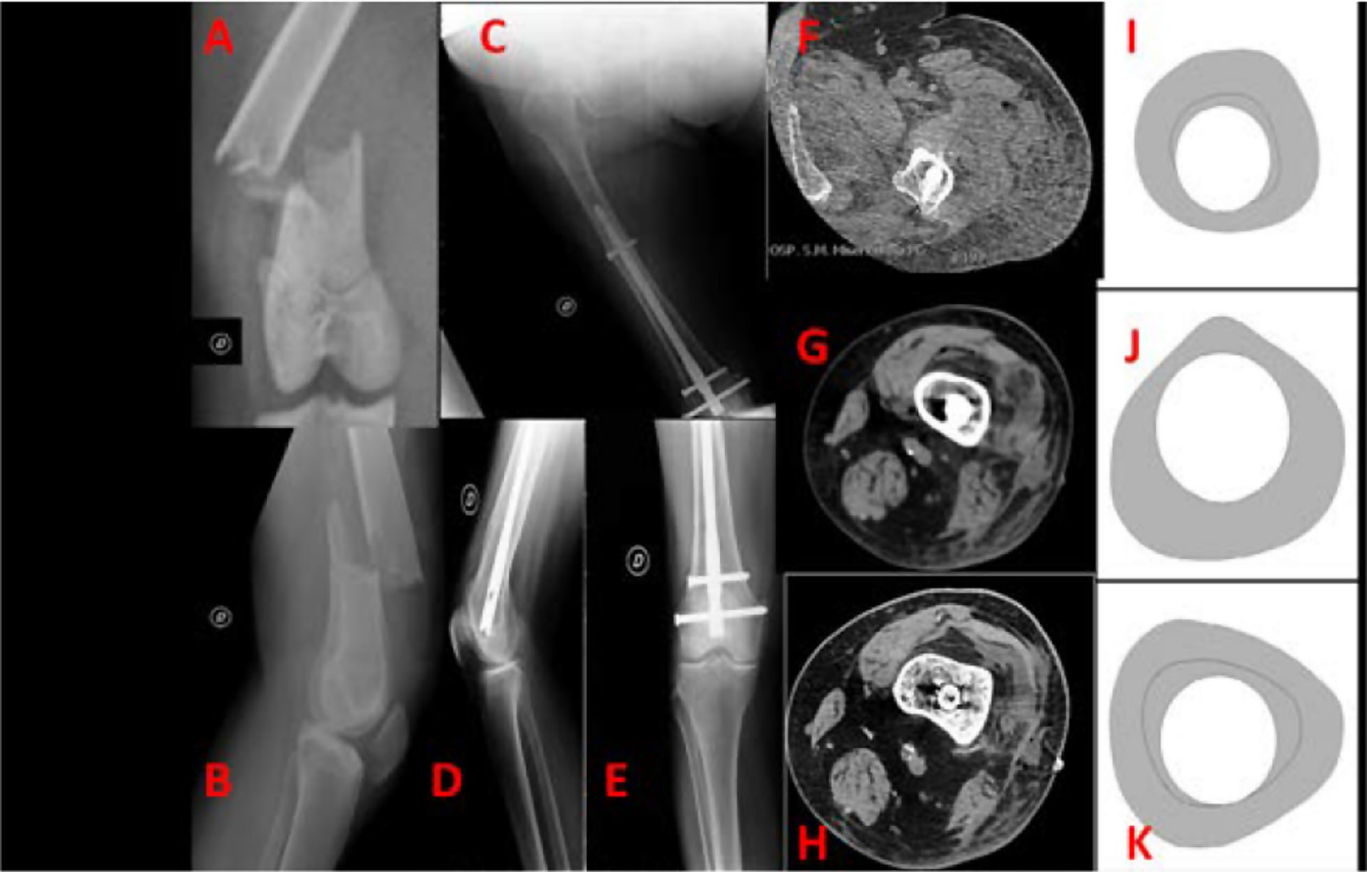
47F, road car accident, 32 A3 type of fracture (**A**, **B**). X-ray at 12 months post-operation (**C**–**E**) showed satisfactory fracture healing after locking retrograde nail. Absence of stress shielding of the nail is shown in **F**–**H** comparing images with biomechanics representations (**I**–**K**) proposed by Shih et al.

**Fig. 3:**
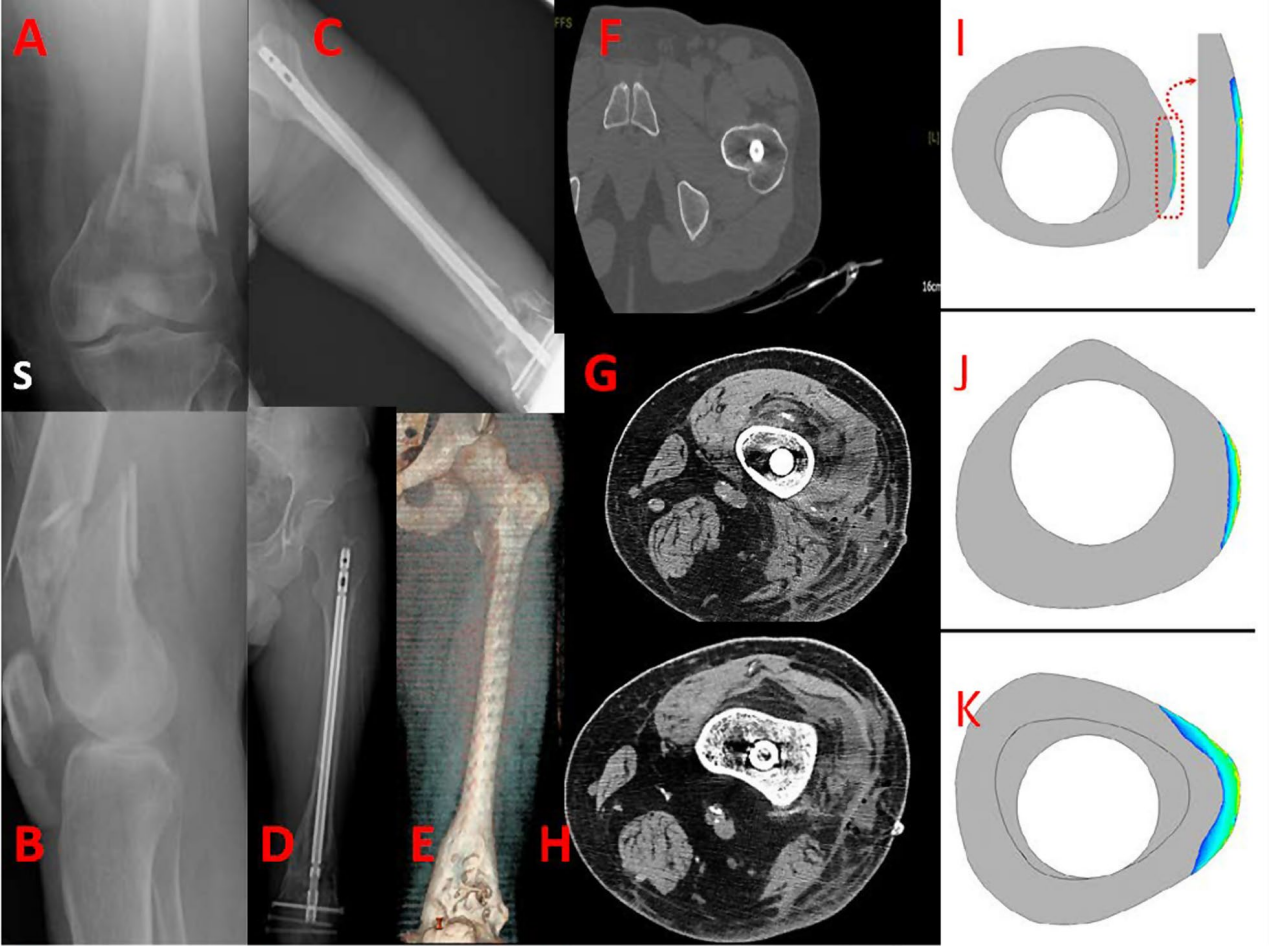
35M, fall from height, 32 C3 type of fracture (**A**, **B**). Post-reduction X-ray with fixation with non-locking retrograde nail (**C**). X-ray and computed tomography (CT) scan at 12 months post-operation show satisfactory fracture healing (**D**, **E**). Absence of stress shielding of the nail is shown in **F**–**H** comparing images with biomechanics representations (**I**–**K**) proposed by Shih et al.

Again similar results were obtained with regard to bone healing timing. No statistical difference was noted among the groups, with average time of bone healing being 144.5 days for LNR group, 142.6 for NLRN and 143.2 for plate. Similarly, RUSH scores were noted to be not very dissimilar (26.1 for LNR group, 26.5 for NLRN group, 26.3 for plate group). These results support the hypothesis that good fracture healing is achieved with any of the three studied surgical techniques and that bony healing is not negatively affected by any of the same procedures. (Figs. 2, 3).

**Fig. 4:**
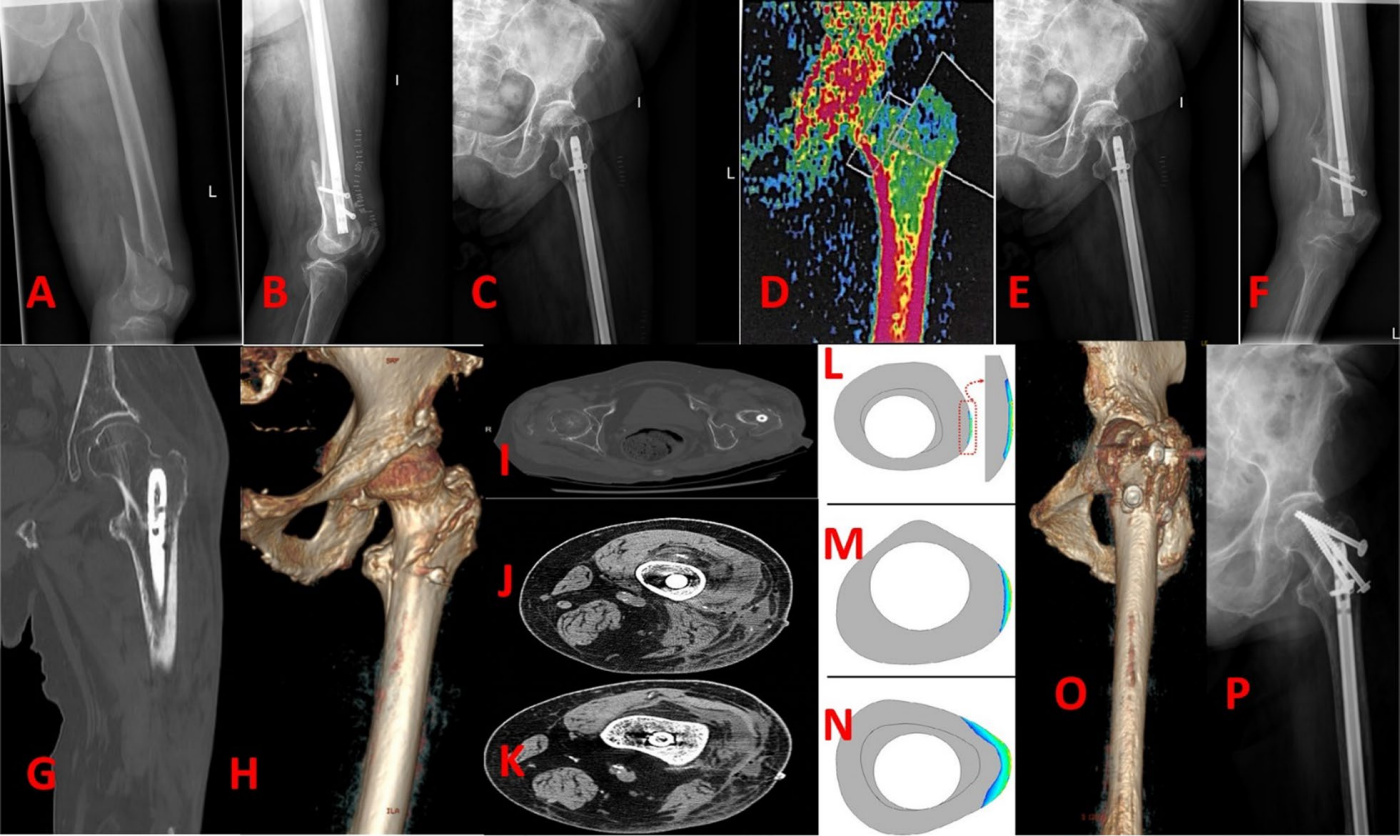
Forty-eight-year-old female patient sustaining a distal third femoral fracture (**A**). Surgical treatment of the same patient with blocked retrograde intra-medullary nail (**B**, **C**). DEXA scan 1 year after surgery showing osteoporosis at the level of the trochanters and femoral diaphysis (**D**). Femur X-ray 1 year after surgery shows femur demineralization of the proximal femoral third (**E**) and complete fracture union at the distal femoral third (**F**). Two months after the latest follow-up visit, the patient presented to the local hospital after a fall, complaining of ipsilateral hip pain. Femur X-ray and CT scan highlighted an undisplaced intertrochanteric fracture (**G**, **H**). CT scan cuts at the level of the greater trochanter (**I**), isthmus (**J**) and condyles (**K**) are compared with biomechanical studies belonging to Shih et al. [22] (**L**–**N**) and show shielding of the nail with stress applied to the intertrochanteric area. The intertrochanteric fracture was surgically managed with three cannulated screws. 6 months post-operation X-ray and CT scan showed fracture union (**O**, **P**)

We recorded only one case of late complication of a 48-year-old female patient from the LRN. She exhibited osteoporosis at the 1 year post-operation DEXA scan and sustained an ipsilateral undisplaced intertrochanteric fracture. The fracture was successfully treated with three cannulated screws, and the 6 months post-operation X-ray showed fracture union. This patient achieved good recovery and mobility status (Fig. 4).

A questionnaire was administered at the time of bone fracture healing shown on X-rays. Similar VAS scores were obtained in all groups (2.4 in LNR group, 2.3 in NLRN group, 2.2 for plate group), in keeping with our hypothesis. Linking RUSH and VAS scores, we found that the regression between RUSH and VAS scores showed a *p*-value of 0.057 in LNR, a value of 0.062 in NLRN and a value of 0.061 in plate. More strength and significance to our results is given by Cohen *κ* values for the average correlation of clinical–radiographic results and patients outcomes: *κ*: 0.859457333 ± 0.08510 for LNR, *κ*: 0.823026667 ± 0.09557 for NLRN and *κ*: 0.853606667 ± 0.06078 for plate.

Correlating clinical outcomes of the groups with radiographic outcomes, we could not find any statistical difference (*p* < 0.05) among the two aspects at the 12 months post-operation follow-up. We considered this similarity as a further strong factor highlighting the absence of significant statistical differences among the studied surgical procedures. This is further supported by the fact that significant difference in terms of bone densitometry was not found in the groups following evaluation of DEXA scans results. Only one patient of the LRN group was found to have a reduction of mineral bone densitometry values at the evaluation endpoint.

We believe that the obtained radiological results are strong, objective and undebatable findings able to support our hypothesis. In fact, clinical and functional results can potentially be biased by subjective factors linked to the patients (age, sex, comorbidities, compliance to rehabilitation, personal goals). In contrast, radiological findings are only marginally influenced by the same subjective factors, and the homogeneity of the obtained radiological results seems to be able to provide quite solid information in support of our theory and aim of the study.

To the best of our knowledge, this is the first study that correlates the above results for patients with distal third femoral shaft fractures treated with LNR, NLNR or plate. Thus, we are not able to compare our results with those obtained in other studies, given the already mentioned lack of similar works. We believe that our results could be relevant for the orthopaedic surgeons dealing with distal femur fractures by providing the interesting tip that the use of LNR, NLNR or plate could not significantly affect clinical, functional or radiological results. This means that decisions must be taken respecting the traditional already accepted indications but also considering the experience and consensus of the surgical team (and multidisciplinary team when necessary: for example, patients with significant comorbidities, polytrauma patients or high risk of mortality following surgery). Surgeons can therefore individualize their approach and technique choice on the basis of patients’ characteristics and comorbidities, pre-injury function, mobility status and rehabilitation pace goals. This could allow the achievement of better individualized results based on individual patients’ potentialities rather than on more standardized algorithms, which take less into account specific objective patients’ factors. The possibility to achieve good outcomes with any of the studied procedures can also be seen as a safety net for all orthopaedic surgeons, particularly the youngest and least experienced, as it seems that they can pick the procedure they are more experienced in (if allowed by the indications) without the fear of compromising the final outcomes.

Therefore, we would like to reinforce the final message that the studied procedures provide good subjective and objective results as low complication rates, good union timing and similar satisfactory experience for the patients have been recorded. More objective and/or subjective outcomes may be also studied in adjunct to the available ones in order to have a wider scenario and stronger results. In fact, the paucity and variable results currently present in the literature do not allow generalization and definitive validation of our results. Furthermore few studies have shown different results and even statistical differences among the techniques, but lack of high level of evidence does not allow us to comment or judge on these studies [18]. We advocate the need for a more powered study and bigger cohorts in order to definitively validate (or eventually reject) our hypothesis. Recent reviews highlighted too the major limitations of the available evidence concerning current treatment interventions for fractures of the distal femur exist and that the currently available evidence is incomplete and insufficient to inform current clinical practice [25, 26]

## Conclusions

No statistical difference was found in our study among the three groups in the treatment of distal third femoral shaft fractures with regard to both objective and subjective outcomes. This allow surgeons to choose among the studied procedures, basing their preference on their experience and individualized indications based on specific objective patients’ characteristics.

The absence of relevant similar data in the published literature does not allow definitive validation (or rejection) of our hypothesis. A more powered study with bigger cohort is needed for definitive validation.

## Data Availability

The datasets used and/or analysed during the current study are available from the corresponding author on reasonable request.
